# EM Structure of the Ectodomain of Integrin CD11b/CD18 and Localization of Its Ligand-Binding Site Relative to the Plasma Membrane

**DOI:** 10.1371/journal.pone.0057951

**Published:** 2013-02-28

**Authors:** Brian D. Adair, Jian-Ping Xiong, José Luis Alonso, Bradley T. Hyman, M. Amin Arnaout

**Affiliations:** 1 Structural Biology Program, Massachusetts General Hospital and Harvard Medical School, Charlestown, Massachusetts, United States of America; 2 Leukocyte Biology and Inflammation Program, Massachusetts General Hospital and Harvard Medical School, Charlestown, Massachusetts, United States of America; 3 Division of Nephrology, and Institute for Neurodegenerative Disease, Massachusetts General Hospital and Harvard Medical School, Charlestown, Massachusetts, United States of America; University of Bergen, Norway

## Abstract

One-half of the integrin α-subunit Propeller domains contain and extra vWFA domain (αA domain), which mediates integrin binding to extracellular physiologic ligands via its metal-ion-dependent adhesion site (MIDAS). We used electron microscopy to determine the 3D structure of the αA-containing ectodomain of the leukocyte integrin CD11b/CD18 (αMβ2) in its inactive state. A well defined density for αA was observed within a bent ectodomain conformation, while the structure of the ectodomain in complex with the Fab fragment of mAb107, which binds at the MIDAS face of CD11b and stabilizes the inactive state, further revealed that αA is restricted to a relatively small range of orientations relative to the Propeller domain. Using Fab 107 as probe in fluorescent lifetime imaging microscopy (FLIM) revealed that αA is positioned relatively far from the membrane surface in the inactive state, and a systematic orientation search revealed that the MIDAS face would be accessible to extracellular ligand in the inactive state of the full-length cellular integrin. These studies are the first to define the 3D EM structure of an αA-containing integrin ectodomain and to position the ligand-binding face of αA domain in relation to the plasma membrane, providing new insights into current models of integrin activation.

## Introduction

Integrins are non-covalent αβ heterodimeric cell adhesion receptors that regulate diverse biological processes by signaling bidirectionally across the plasma membrane (reviewed in [1]). Crystal structure of αVβ3 [2] and αIIbβ3 ectodomains [3] revealed that integrins adopt a compact bent conformation that consists of a ligand-binding ‘head’ comprising a seven-bladed Propeller domain from the α-subunit bound noncovalently to a vWFA domain (βA domain) from the β-subunit. The Propeller domain is followed by a Thigh- and Calf-1 and Calf-2 domains, a transmembrane (TM) segment ending with a short cytoplasmic tail. βA is inserted in an Ig-like Hybrid domain, which is flanked by an N-terminal PSI domain and followed by four EGF-like domains (EGF1-4), a beta-tail domain (βTD), a TM segment and terminating with a short cytoplasmic tail. Bending in the structure occurs at two knees (genu) (located between the Thigh and Calf-1 domains of the α-subunit and in EGF2 of the β-subunit [4]). Mg^2+^-dependent binding of physiologic ligands takes place at the integrin head, and requires a *m*etal-*i*on-*d*ependent-*a*dhesion-*s*ite (MIDAS) in the βA domain [5]. In nine of the twenty-four mammalian integrins, an additional vWFA domain (αA domain) is inserted in the Propeller domain, and acts through its MIDAS face as the principal ligand-binding site [6].

The αA-containing leukocyte integrin CD11b/CD18 (αMβ2) is the major integrin expressed in phagocytic cells, and plays a vital role in host defense by mediating the extravasation of phagocytic cells to inflamed organs and the phagocytosis of serum-opsonized pathogens [7]. Like most integrins, CD11b/CD18 is normally expressed on the cell surface in an inactive conformation, thus allowing leukocytes to circulate in the blood stream and preventing pathologic inflammation. Previous studies in other integrins have elucidated an essential role for hydrophobic and electrostatic interactions between the α- and the β-subunit TM/cytoplasmic tails (reviewed in [8]) in stabilizing the inactive state. These interactions have been mimicked experimentally by placing an artificial inter-subunit disulphide in the membrane proximal α/β linker regions of the ectodomain [4], [9], [10]. In response to activation signals, the cytoskeletal protein talin disrupts the integrin cytoplasmic α/β interaction [11], inducing an allosteric rearrangement that shifts the conformational equilibrium of the ectodomain in favor of the ligand-binding state, a process that may also require kindlins [12]. The crystal structure of the isolated αA domain (CD11bA) from integrin CD11b/CD18 has been determined in its inactive [13] and ligand-occupied states [14], distinguished by local structural changes that modulate the ligand-binding competency of MIDAS. Ligand-bound αA acts as an intrinsic ligand for the βA domain [15], triggering the conformational changes that lead to outside-in cell signaling. Crystal structure of CD11bA in complex with the ligand-mimetic Fab fragment of mAb107, a Ca^2+^-mediated interaction that stabilizes CD11bA in the inactive conformation, has been recently derived [16], opening new venues for modulating the functional activity of this integrin. However, no information on the 3D structure of the CD11b/CD18 ectodomain or the position of its CD11bA relative to the cell membrane is available to date. A crystal structure of a related CD11c/CD18 ectodomain in its bent inactive conformation has been recently reported [17]. Since only two out of ten molecules in that structure were defined, it was concluded that the αA domain is very flexible within the integrin head.

In this communication, we used electron microscopy and 3D image reconstruction to determine the 3D structure of inactive CD11b/CD18 ectodomain as well as its EM structure bound to the Fab 107. Surprisingly, we find that CD11bA is restricted to a relatively small range of positions within a bent ectodomain conformation in the 3D EM structure. Using high-resolution fluorescent lifetime imaging microscopy (FLIM) in full length CD11b/CD18, we show that CD11bA is positioned well beyond the membrane surface in the inactive state, while a systematic orientation search defines a specific tilt angle between the ectodomain and TM domains, which would allow access of the inactive integrin to soluble ligand. The significance of these findings in relation to current models of integrin activation is discussed.

## Materials and Methods

### Reagents and antibodies

Restriction and modification enzymes were obtained from New England Biolabs Inc. (Beverly, MA). The cell culture reagents were purchased from Invitrogen Corp (San Diego, CA) or Fisher Scientific (Hampton, NH). The β2 integrin heterodimer-specific murine mAb IB4 [18] and the ligand-mimetic mAb107 [19] have been previously described. The fluorescent labeling reagents Alexa-488 NHS ester and FM 4-64FX were from Invitrogen Corp. The Fab-fragment (Fab107) of mAb 107 IgG was prepared by papain digestion followed by affinity chromatography as described [16]. Fab107 was labeled with Alexa Fluor 488 N-hydroxy succinimidyl ester (NHS) dye using the manufacturer's recommended protocol and excess dye was removed using Centri-Spin 20 size-exclusion microcentrifuge columns (Princeton Separations, Adelphia, NJ).

### Cell culture and transfection

Stable transfection of wild-type (WT) CD11b/CD18 or the same integrin containing the activating I316G mutation in CD11bA [16] was carried out in K562 cells (from American Type Culture Collection, Manassas, VA) using published protocols as previously described [20]. Transformed cells were maintained in IMDM supplemented with 10% heat-inactivated fetal bovine serum, 100 IU/ml penicillin, 100 µg/ml streptomycin and 0.5 mg/ml G418.

### Flow cytometry

Harvested K562 were washed in Hepes-buffered saline (50 mM Hepes, 150 mM NaCl, pH 7.4) containing 0.1% BSA (washing buffer, WB), incubated in 10 mM EDTA in WB for 5 min at room temperature, then washed in WB three times. 1×10^6^ cells were suspended in 100 µl WB containing either 1 mM CaCl_2_ or 5 mM MgCl_2_/1mM EGTA (which converts β2 integrins into ligand-competency [21]) for 10 min at 37°C, then stained with mAbs 107 or IB4 (each at 20 µg/ml, for 30 min at 37°C). After washing with the respective metal ion-containing buffer, cells were incubated with allophycocianin-conjugated goat anti-mouse Fc specific antibody (Jackson) at 1∶30 dilution in the dark at 4°C for another 30 min. Washed cells were resuspended in 400 µl of the respective metal ion-containing buffer, fixed in 2% paraformaldehyde and analyzed by flow cytometry in a FACSCalibur flow cytometer (BD Biosciences) counting 20,000 events. Mean fluorescence intensity (MFI) was determined using the CellQuest software (BD) and binding of mAb 107 was expressed as a percentage of mAb IB4 binding.

### Expression and purification of CD11b/CD18 ectodomain

Cloned cDNA encoding human CD11b ectodomain (residues F1-N1088) and containing the I316G substitution in CD11bA were fused in-frame and upstream to a cDNA sequence encoding a single cysteine residue (replacing P1089 in CD11b) that is followed by a PreScission protease site, an Acid coiled-coil motif and a 6-Histidine tag. cDNA encoding human CD18 ectodomain (residues Q1-N678) was similarly inserted into a cDNA sequence encoding a single cysteine residue (replacing I679 in CD18), a PreScission protease site and a Basic coiled-coil motif. Both constructs were cloned into a pCF expression vector. The resulting positive clones were used to transfect glycosylation-deficient CHO Lec 3.2.8.1 cells (kindly provided by Dr. Pamela Stanley, Albert Einstein College of Medicine, NY)[22] using Lipofectamine 2000, to make stable cell lines under double selections (G418 and Hygromycin). Protein expression was monitored using ELISA, in which mAb IB4 was used as the catch antibody, and anti-His antibody as the secondary antibody. The clones giving the highest expression level were kept for protein production in triple layer flask. Secreted protein was first purified on Ni-NTA affinity column, then using mAb107 affinity column. After cleavage of both ACID-BASE coiled-coil motifs and His-tag, the digested protein was further purified on Superdex 200 10/30 HR size-exclusion column. A yield of purified heterodimeric protein of ∼0.5 mg/L of cell culture was obtained. SDS-PAGE analysis under non-reducing conditions revealed a single Coomassie-stained band corresponding to the disulphide-linked heterodimer, which converted to two bands corresponding to the CD11b and CD18 subunits under reducing conditions (not shown).

### Electron microscopy and image analysis

Aliquots (∼5 µl) were allowed to adhere for ∼30 sec to carbon-coated copper grids that had been rendered hydrophilic by glow discharge in air and then stained with 0.75% uranyl formate (Electron Microscopy Sciences, Hatfield PA). Images were recorded under minimum electron dose conditions using a CM100 electron microscope (Philips Electron Optics/FEI, Eindhoven, The Netherlands). Images were recorded on Kodak SO163 film at a nominal magnification of 35,000 for CD11b/CD18 ectodomain or 46,000 for the integrin/Fab107 complex, using 100 kV electrons. Micrographs were digitized with a Super Coolscan9000 ED scanner (Nikon, Inc.) at 8 bits per pixel and 6.35 µm per pixel, subsequently averaged to 12.7 µbm per pixel. The optical density for each negative was adjusted to give a mean value of ∼127 over the total range of 0 to 255. Image processing was performed with the EMAN suite. For the CD11b/CD18 ectodomain, an automated, model-free particle selection routine implemented in the EMAN program *boxer* was used to select a total of 8,825 particles from 12 negatives. CTF parameters for each negative were determined with the EMAN program *ctfit* and phase corrections performed for each particle. Reference-free class averages were generated with the routine *startnrclasses*, followed by seven rounds of reclassification using the projection matching routine *classesbymra*, as implemented in the script *refine2d.py.* 3D refinements were performed with the EMAN routine *refine* using a 10° angular spacing for model projections. Initial models were generated from pdb files using *pdb2mrc*. The convergence of the refinements was monitored by examining the Fourier shell correlation between successive models during the refinement and halted when the 50% correlation position failed to show any improvement. Estimation of the resolution of final models was performed by examination of the Fourier shell correlation between models produced by separating the raw particles into two groups and calculating separated 3D maps from each group using the classification generated from the final round of refinement. For the integrin/Fab107 images, the same methods were used to select 10,661 from 20 negatives. Reference-free class averages were generated from the CTF corrected particle set identically as with the CD11b/CD18 data set.

### FLIM Assays

Wells of non-fluorescent Labtek II four-chamber microscope slides (Nalgen Nunc, Rochester, NY) were coated with poly-L-lysine (Sigma-Aldrich St. Louis, MO) overnight at 4°C. CD11b/CD18 K562 cells (25,000–50,000) were transferred in Tris-buffered saline, pH 7.4 (TBS) to each well and incubated for one hour at 37°C in a total volume of 200 µL. Non-specific sites were then blocked by incubation with TBS containing 1% Bovine Serum Albumin (Sigma-Aldrich) for 30–60 min at 37°C. The wells were washed twice with TBS to remove non-adherent cells. The remaining cells were preincubated in TBS containing 1 mM Ca^2+^ (TBS^+^) for 15 min at 37°C. For immunolabeling, adherent live K562 cells stably expressing CD11b/CD18 were stained with 20 µg/ml Fab107-Alexa488 in TBS^+^ for 30 min at 4°C. After washing twice with TBS^+^, and once in Hank's Balanced Salt Solution containing 1 mM Ca^2+^ (HBSS^+^, Invitrogen), Fab107-Alexa488- labeled cells were incubated in the absence or presence of 6–12 µM FM solution in HBSS^+^ for 10 min at 4°C, washed in HBSS^+^, then fixed with ice-cold 4% paraformaldehyde in PBS for 10 min at 4°C, washed with ice-cold PBS and mounted with GVA mount (Invitrogen) under a cover slip. The GVA-mounted slides were kept overnight in the dark at room temperature to set the mounting media and used the next day in FLIM assays.

FLIM assays were performed as previously described [4]. Briefly, a mode-locked Ti-sapphire laser (Spectra-Physics, Fremont, CA), which sends a femto-second pulse every 12 nsec to excite the fluorophore at 800 nm, was used for all assays. Images were acquired using a Bio-Rad Radiance 2000 multi-photon microscope (Hercules, CA). A high-speed photon detector (MCP R3809, Hamamatsu, Bridge water, NJ) coupled to time-correlated single photon counting SPC830 hardware and SPCImage software from Becker and Hickl (Berlin, Germany) was used to measure fluorescence lifetimes on a pixel-by-pixel basis. This set-up allows high temporal resolution lifetime acquisition with high spatial resolution imaging. Laser power was kept fixed at 2%. Data from individual cells was collected in each condition. A 128×128 pixel matrix was created with the single exponential (for donor alone) or multi-exponential (donor in the presence of acceptor) curve-fit data for each pixel, allowing color-coding by lifetime over the entire image. As a negative control, Alexa488 lifetime was measured in the absence of the acceptor (donor only). Percent lifetime decrease, or FRET efficiency, E, was calculated as the difference between excited state of the donor in absence (τ_D_) and in presence (τ_DA_) of acceptor fluorophore, or: E = 1−τ_DA_/τ_D_. The distance between donor and acceptor fluorophores, r, was calculated using the following equation [23]: r = R_0_ (τ_D_/τ_DA_ −1)^−1/6^, where R_0_ is the Förster radius, or the distance at which energy transfer is 50%. Here, we used a value of 62Å, estimated based on the Forster radius of Alexa Fluor 488 with Alexa Fluor 568 as acceptor (Invitrogen website).

### Orientation Modeling

The structure of the CD11b/CD18 in complex with Fab107 was derived from the reference-free class averages from EM images of the complex by sequentially determining the orientation of the integrin ectodomain (without CD11bA) for each average followed by the determination of the CD11bA/Fab107 orientation. An electron density map from the αVβ3 ectodomain filtered to 18Å resolution was generated with the EMAN routine *pdb2mrc* and 2D projections generated at 8° intervals with *project3d*. The projections were searched against the class averages with the EMAN program *classesbymra2* to determine the Φ and Θ Euler angles. The remaining parameters (x and y shifts, Euler angle ω) were determined by a cross-correlation search of the reference projection with the class average. The aligned reference projection was subtracted from the class average and the resulting difference map classified against a set of reference projections prepared from the CD11bA/Fab107 structure. Additional models were generated with the CD11c/CD18 ectodomain X-ray structure (pdb id 3K71), using chains G and H that possess the αA domain density. Homology modeling between the two αA domains was used to place Fab107 on the CD11c/CD18 structure. To generate a canonical ‘bent model’, crystal structure of inactive αVβ3 ectodomain (pdb 1jv2) was superimposed on that of CD11c/CD18 ectodomain by homology modeling between the α-chain Propeller domain while the Fab107/CD11bA structure was superimposed on CD11c/CD18 as before. The final model was generated by deleting the CD11c/CD18 chains leaving the oriented αVβ3 ectodomain/Fab107 CD11bA chains. Each of the models was oriented to a common origin by a series of rigid body rotations and translations. A vector to define the Calf1/Calf2 domains was chosen by setting one end point to the center of the disulfide bond defining the large loop at the end of Calf-1 (C755 and C761 in CD11c; C596 and C602 in αV) and the other end point as the last residue in α-subunit (P1090 in CD11c; W953 in αV). The last residue in the α-subunit was translated to coordinate 0,0,0 in space and the model rotated to position the vector vertically (x = y = 0). To systematically generate different orientations, the models were first rotated about the z axis (angle Φ), then rotated around the y axis (angle Θ). To determine clashes with the membrane the coordinates of each atom were compared with an x-y plane representing the membrane. The z-coordinate of the plane was positioned at the start of the transmembrane helix, and the distance between the helix start and the last residue in the α-chain used the previously determined model of the intervening residues in αV [4]. Due to the random labeling of Fab 107 and the large number of individual Fab molecules on the cell, the average distance is given by the centroid for the fluorophores. To derive a single point to represent the Fab, the centroid between the α-carbons of all of the lysine residues in the in the Fab107 X-ray structure [16] was therefore used as previously described [4].

## Results

### Electron microscopy analysis indicates that the isolated CD11b/CD18 ectodomain is in a bent conformation

**Figure 1:**
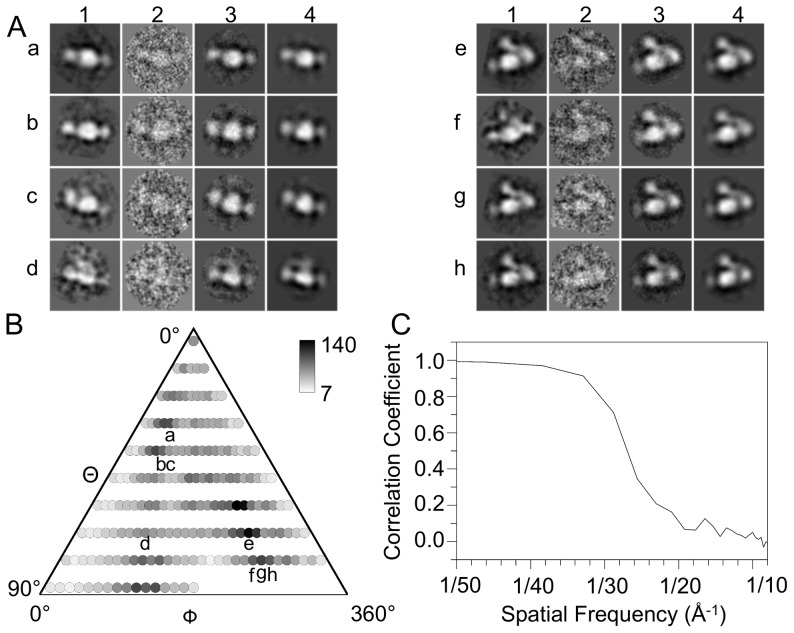
EM refinement of the CD11b/CD18 ectodomain. (A) Example images from the final round of refinement. On the left is an average image generated from reference-free alignment and averaging which preceded the refinement itself. The averages have been displayed with the final map projection that they most closely resemble. Results from the final round of refinement are displayed adjacent to the reference-free average, showing from left to right of each panel the average from reference free alignment (number 1), an example raw particle from the final projection class (number 2), the average generated from the final projection class (number 3), and the projection from the final map for this class (number 4). The letters on the left indicate the class in the distribution histogram (B). The side of each box is 232Å. (B) Projection histogram showing the number of particles identified as belonging to a particular Euler angle orientation for the final round of refinement. Each circle indicates a particular class while the number of particles is indicated as a gray scale. The scale bar is indicated to the right. The letters refer to the classes shown in (A). (C) Fourier shell correlation analysis to determine the resolution of the final map. The total dataset was separated randomly into two equal groups. The particle classes for each group were independently aligned with the final map projection and averaged to generate two independent maps. The graph displays the correlation in Fourier space between the maps. The resolution of 1/(26 Å) is determined by a value of 50% correlation.

We determined the 3D EM structure of CD11b/CD18 ectodomain in buffer containing the inhibitory Ca^2+^ [24], which stabilizes the inactive state. The full dataset contained 8,825 particles selected from negatively stained micrographs using an automated, model-free algorithm. Preliminary analysis was conducted with reference-free alignment and classification, where raw particle images are aligned to a common center and classified using features extracted from the covariance of the global average. This method, which does not rely on any presupposition of the protein's structure, produced several averages, which closely resemble views expected from a bent conformation of the ectodomain (Figure 1). Indeed, all of the resulting averages were compact, and the majority could be assigned to a bent conformation. Side views displayed clear density for CD11bA domain, indicating that in CD11b this domain is relatively stable, as conformational flexibility would have led to its density being averaged out.

3D image analysis and reconstruction bore out the bent conformation for the CD11b/CD18 ectodomain. The 3D analysis was performed using the full dataset (8,825 particles) employing a projection-matching algorithm implemented in EMAN. The model generated in the final round included 7,587 particles from the data set. The remaining particles were rejected by the algorithm as a result of statistical tests during the refinement. The rejected particles may represent CD11b/CD18 in alternate conformations, denatured protein, poorly stained particles or contain only background. The resulting 3D structure closely resembles a low-resolution structure calculated from the αVβ3 ectodomain [25] (with an added αA domain), rather than the related CD11c/CD18 ectodomain crystal structure [17]. Results from the final reconstruction are shown in Figure 1. Although each of the 181 projection classes contained particles, the raw particles did show a preferential orientation for only a few Euler angles (Figure 1B). These views showed the integrin ectodomain in a characteristic side view, with a clear ‘V’ shape formed by the angle between the Thigh domain and the Calf-1/Calf-2 domains, or rotated by 90° such that the CD11b Propeller and Thigh domains are facing the viewer (Figure 1A, a, b). This model rapidly converged, and after the sixth round subsequent rounds of refinement did not substantially change the structure or improve resolution. Resulting averages and projections from some of the classes in the final round are shown in Figure 1A. As can be seen, the final class averages closely resemble the map projections. Also shown are example raw particles to demonstrate the agreement between particles and the averages, and some of the reference-free averages, which were calculated without any model input. The reference-free averages have been placed near the final class average that they most closely resemble. The final map faithfully represents the protein conformation as found in the dataset, and is not the result of model bias. The final resolution of the map as determined by Fourier shell correlation is ∼26Å using the 50% cutoff criterion (Figure 1C).

**Figure 2:**
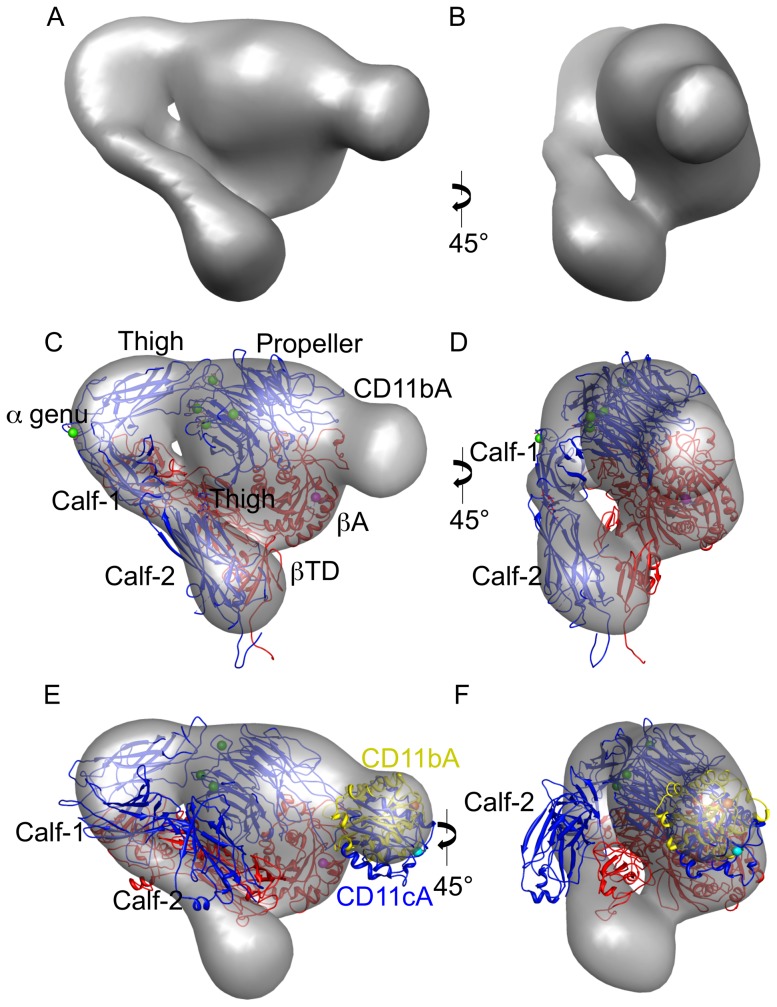
Surface shaded density of the final 3D map of the CD11b/CD18 ectodomain. A, B) are two views of the map rotated by 45°. The isosurface has been chosen to enclose 100% of the expected protein volume. C and D) the same views of the map as in A and B, shown as a transparent isosurface, but also displaying the αA-lacking αVβ3 ectodomain (pdb 3IJE) model as a ribbon diagram. A clear density corresponding to the CD11bA domain is seen, fitted here with a ribbon diagram of the crystal structure of the isolated CD11bA (pdb 1jlm), with the MIDAS ion in cyan. The αV subunit and CD11bA are shown in blue and β3-subunit in red. The spherical metal ions in the Propeller and α-genu are in green and that in the βA ADMIDAS is in magenta. E, F) a ribbon diagram of the crystal structure of CD11c/CD18 ectodomain fitted into the CD11b/CD18 EM map (shown as a transparent isosurface). Most of Calf-1/Calf-2 and β TD domains do not fit the 3D map. CD11cA fits better within the map density but extends somewhat outside it (compare CD11cA with that of CD11bA [shown in yellow, oriented as in C, with its MIDAS metal ion in orange]). The three metal ions in the CD11c Propeller are in green, the CD11cA MIDAS ion in cyan, and the βA ADMIDAS ion in magenta. F) a 45° clockwise rotation of the Figure in (E).

The resulting map from the final round of refinement is shown in Figure 2A–D. The map has been filtered to 26Å, and the isosurface set to enclose 100% of the expected protein volume. Excluding the density attributable to the CD11bA domain, the map resembles the canonical ‘bent’ conformation as seen in the αVβ3 ectodomain structure [25]. The map density does not fit well the X-ray structure for CD11c/CD18 ectodomain (Figure 2E, F): While the head, Thigh and Hybrid domains fit well into the map density, Calf-1/Calf-2 clearly lie outside it (Figure 2E, F). Both ectodomains contain an engineered interchain disulfide bond at the identical position in the membrane-proximal α/β linker region of the ectodomain, so the difference is more likely the result of crystal contacts among the four molecules in the unit cell of CD11c/CD18 ectodomain distorting these domains from their position in solution. The αA domain from the CD11c structure fits better within the map density but extends somewhat outside it (Figure 2E, F), requiring a small 5Å translation from its position in the CD11c X-ray structure [17].

### Determination of the relative orientation of the MIDAS face of CD11bA domain in the integrin ectodomain

**Figure 3:**
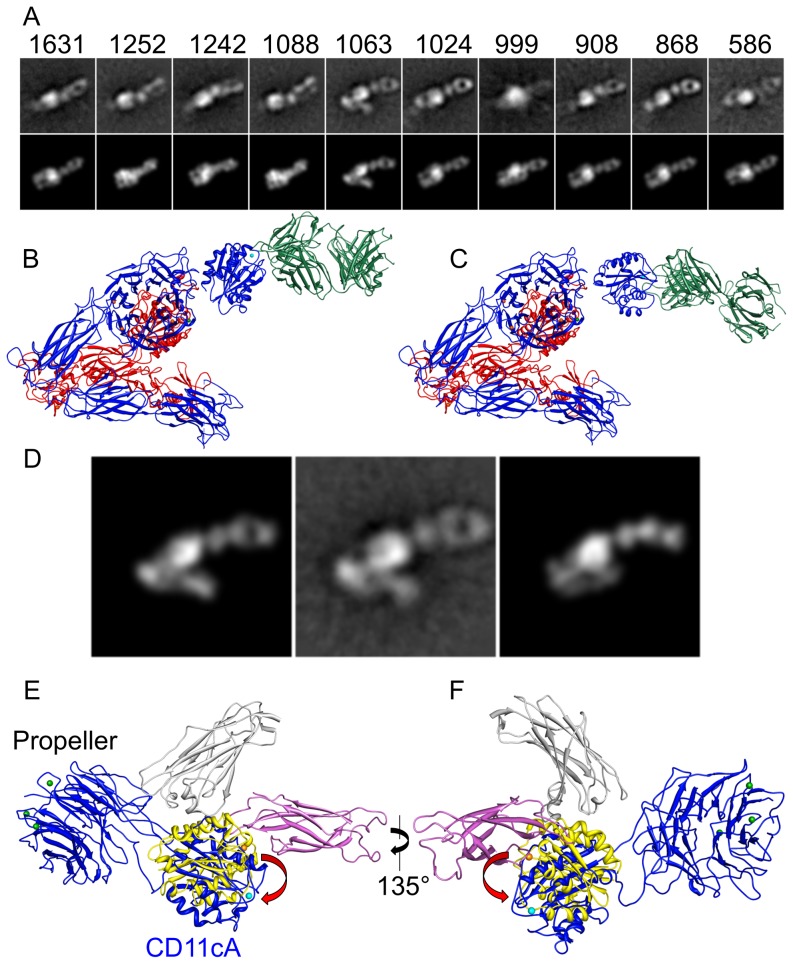
EM analysis of the CD11b/CD18-Fab107 complex and orientation of the CD11bA MIDAS face. (A) Reference-free alignment and averaging of CD11b/CD18-Fab107 particles. The top row shows a k-means classification of 10,661 raw particles classified into 10 groups and averaged. The number of particles for each class is indicated at the top of each average. Several of the groups are similar, suggesting that there are fewer than 10 unique preferred orientations for the particles on the grid. The bottom row shows the model projection that best fits each of the particles shown in the top row. Projections in the bottom row were generated from the model shown in (B) which was generated by sequentially fitting a projection for the integrin followed by the projection for the CD11bA/Fab107 complex. For comparison, the model generated by fitting the CD11c/CD18 ectodomain X-ray structure is shown in (C). In this model, the CD11bA domain has the same relative orientation to the rest of the integrin as found in the X-ray structure. (D) Shows a direct comparison of the two models for the average, which most easily allows identification of individual integrin domains within the projection. Displayed alongside the average (center image) is the projection from the model in (B) which best fits the average (left image), compared to the projection from the model in (C) (right image). E, ribbon diagram of the Propeller/αA of CD11c/CD18 ectodomain crystal structure oriented as in Figure 2E, showing crystal contacts from two symmetry-related molecules (Calf-2 in gray and Thigh in magenta) that rotate MIDAS face of CD11cA domain by ∼35° (red arrows) relative to its position in the unrestricted EM model (colored as in Figure 2E). F, same Figure in (E) rotated clockwise by 135°.

Because of the low resolution of our 3D EM map and the generally spherical nature of αA domain, we sought to independently assess its orientation relative to the Propeller domain by further examining the EM structure of a complex formed between the CD11b/CD18 ectodomain and Fab 107, a ligand mimetic that binds at the MIDAS face and stabilizes the integrin in the inactive state in the presence of the inhibitory Ca^2+^ [16](Figure S1). The Fab fragment binds by an extensive and well-defined interface and thus allows identification of the CD11bA MIDAS face even at low resolution. Class averages generated from these particles (Figure 3A) closely resemble the averages generated from the ectodomain on its own, with the exception of a bilobed density attributable to the Fab. This indicates that the ectodomain within the complex is also in the bent conformation and that the Fab does not seem to interfere with the preferential orientation of the integrin on the grid. The presence of well-defined Fab density indicates that CD11bA/Fab107 complex possesses a limited range of orientations within the integrin, as a highly flexible link between CD11bA and the Propeller would result in the density for the former being averaged out. Attempts to generate a 3D map from the raw particles failed, with the resulting map not being interpretable as either integrin or Fab density. While this may be due to an underlying conformational heterogeneity, we think this unlikely due to the close correspondence between class averages generated from complexed and non-complexed particles. We rather attribute the different results for the two datasets to the different sizes of the masks used during the course of the reconstruction algorithm. The presence of the Fab required the use of a much larger mask than was needed for CD11b/CD18 ectodomain on its own to include all of the protein density. This necessarily included a larger area of the graphite background during the reconstruction, which combined with the low signal-to-noise ratio for these particles to defeat the 3D reconstruction algorithm. In support, a very tightly masked reconstruction of the ectodomain/Fab107 particles, which cuts off the Fab density resembles the map generated from CD11b/CD18 alone (not shown).

The presence of well-defined density attributable to both integrin ectodomain and Fab107 allowed us to determine the relative orientation by matching projections for both elements. We identified the relative angle for the integrin ectodomain by finding the best match between the class average and a set of projections of the integrin ectodomain, and the relative angle for the CD11bA/Fab107 complex found with the same method. Thus, for each class average, a model may be constructed by combining the underlying atomic structures with the identified orientations. We fit the CD11bA domain separately from the remainder of the integrin due to the findings in the CD11c/CD18 ectodomain structure, which suggested that the connection between αA and the Propeller domains would be flexible [17]. The models generated from fitting the class averages are closely similar, and any one of them fits with all of the class averages (Figure 3A), suggesting that CD11bA is restricted to a relatively small range of orientations. The pseudo atomic model derived from EM image analysis (Figure 3B) places CD11bA in a similar location to its position in the model derived from fitting the CD11bA/Fab107 structure into the CD11c/CD18 X-ray structure (Figure 3C). However, an ∼35° rotation of CD11bA from its position in the latter model is sufficient to fit the data derived from the EM structure of the ectodomain alone. The need for this combined movement is illustrated in Figure 3D, which shows a reference-free class average (center image) in comparison with the calculated projection that best fits the average from the EM model on the left, with the best fitting projection from the CD11c/CD18-derived model on the right. The rotation changes the orientation of the Fab, providing distinct, highly characteristic views, and significantly improves the cross correlation coefficient between the model and the class average from 0.79 (in the CD11c/CD18 structure derived model) to 0.88 (in the CD11b/CD18 EM-derived model). The ∼35° rotation of αA domain in the CD11c/CD18 ectodomain crystal structure vs. the EM structure is likely the result of unfavorable crystal contacts from two symmetry-related molecules (Figure 3E, F).

### Fab 107 defines orientation of CD11bA MIDAS face relative to the plasma membrane in inactive cellular CD11b/CD18

Since mAb107 binds the MIDAS face in the presence of the inhibitory Ca^2+^ [16], [19], it provides an ideal structurally defined FLIM probe to site-specifically label the MIDAS face to evaluate its orientation relative to the plasma membrane in inactive CD11b/CD18 in live cells. FLIM measures the fluorescence lifetime of only the donor fluorophore, is relatively insensitive to variations in donor and acceptor fluorophore concentration and optical pathlength and has picosecond resolution, making it ideal for quantitatively determining proximity of two fluorophores. The efficiency of fluorescence resonance energy transfer (FRET) between the donor–acceptor fluorophore pair is quantitatively described by the Förster equation, where the efficiency of transfer E = R_0_^6^/(R_0_^6^+R^6^), such that there is no appreciable FRET between the pair (and consequently no reduction in donor fluorescence lifetime) at a separation distance of>∼2R_0_ between the donor and the acceptor fluorophores, where R_0_ is a constant depending on the spectral properties and the relative orientation of the two fluorophores [26], [27]. In the FLIM experiments, we used Fab 107 to obtain information about proximity and orientation of CD11bA relative to the cell membrane with higher confidence and accuracy. Fab 107 was chemically fluorescently labeled using 'donor’ Alexa488 NHS ester to generate Fab107-Alexa488. We labeled the Fab107 to various degrees with Alexa488 and determined that the post-labeling Fab107:Alexa 488 ratio of ∼1∶3 provided optimum signal-to-noise ratios in FLIM experiments (data not shown). For selective labeling of the cell membrane with an ‘acceptor’ fluorophore, we chose FM 4-64 FX dye (FM), which rapidly and preferentially inserts into the outer leaflet of a cell membrane, and can be rapidly fixed, minimizing diffusion on the FLIM measurement timescale and structurally defining position of the acceptor.

**Figure 4:**
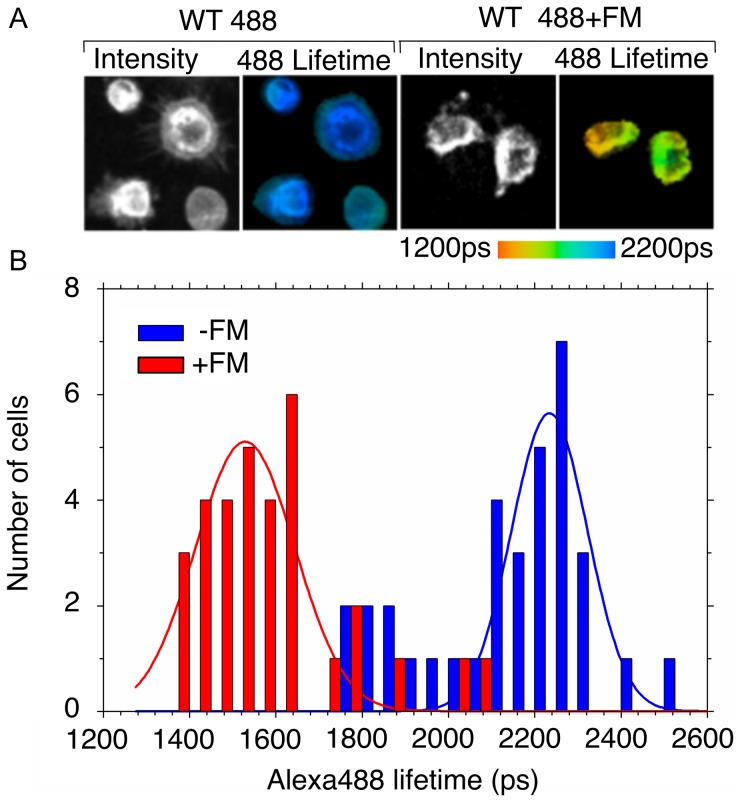
FLIM lifetimes of the Alexa488 donor fluorescence in the presence and absence of the acceptor FM. A) Representative Alexa488 fluorescence intensity and lifetime images. The panel shows examples of data collected in the absence (left, WT 488) and presence of the acceptor FM (right, WT 488+FM). The pseudocolor scale is shown at the bottom. B, histogram of measured Alexa488 lifetimes generated by integrating lifetime measurements for individual cells. The histogram bars show the number of cells in 50 ps bins for Alexa488 in the absence (blue) and presence (red) of the acceptor FM. The average and standard deviation were generated by fitting the histogram to a single Gaussian function (shown as a solid line).

K562 cells stably expressing the recombinant integrin were stained with 20 µg/ml Fab107-Alexa488 at 4°C in a buffer containing 1 mM Ca^2+^ (Figure 4A, B). FLIM measurement in the absence of FM acceptor fluorophore produced a broad range of lifetimes of between 1,651 and 2,513 ps (Figure 4B). When these lifetimes were fit to a Gaussian distribution they were found to possess a mean of 2,234±13 ps (±95% confidence limits) with a standard deviation of 88±13 ps. This is similar to the 2,384 ps lifetime of unbound Fab-Alexa488 (not shown), suggesting that the Fab107-Alexa488 labeled cells are devoid of any endogenous fluorescence acceptor and are fully exposed to the solvent and not interacting with the protein, which might lengthen the lifetime. Solvent exposure indicates that the fluorophores, attached to the ε-amino group of the lysines, are able to tumble freely in solution and thus be randomly oriented. Only a minor population of cells seems to be intrinsically quenched. When the Fab107-Alexa488-labeled cells were further labeled with the membrane dye FM as an acceptor fluorophore, the Alexa488 fluorescence lifetime decreased significantly, generating a range of lifetimes between 1,354 and 2,099 ps. These lifetimes may also be fit to a single Gaussian with a mean of 1,529±14 ps and a standard deviation of 115±14 ps (Figure 4B). There is also a minor population of cells with lifetimes falling outside the fitted distribution, although in this case with longer lifetimes than the mean. In both cases the whole cell lifetimes may be fit to single Gaussian, with correlation coefficients of 0.83 and 0.88 for the quenched and unquenched measurements, respectively. Taking the mean values for the fits to the two datasets yields a FRET efficiency of ∼32% and a mean donor-acceptor separation distance (R) of ∼71Å, using the R_0_(2/3) value of 62Å for this donor-acceptor pair. Using the standard error of the mean produces an error estimate of only±∼1Å. Using±1σ for each of the fitted distributions produces a range of possible R values of between 76Å and 67Å. This result suggests that in the inactive conformation, the MIDAS face of CD11bA in full-length receptor lies in proximity to the cell's lipid bilayer, but not immediately adjacent.

### Modeling CD11b/CD18 ectodomain on the membrane

**Figure 5:**
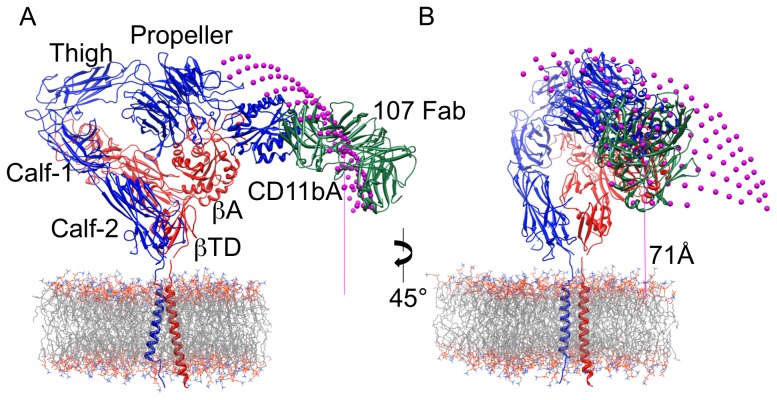
Modeling of the relative membrane orientation of CD11b/CD18 by combining the FLIM and EM data. A) A display of the allowed orientations of the integrin ectodomain. To determine the allowed orientations, the protein is first rotated in the membrane plane and subsequently rotated down. For each rotation pair, the model is evaluated for clashes with the membrane. Shown in magenta are the centroids for Fab107 for all of the resulting angles. For illustration purposes, the integrin α- (blue), β- (red) subunits and Fab107 (green) chains are displayed as a ribbon diagram for one of the resulting orientations. A section of a model DMPC membrane is shown as a wire diagram. The model of the CD11b/CD18 ectodomain is taken from the fit to the EM map. The distance of the centroid for the displayed model is compatible with the measured FLIM distance. B) The same view as in (A) but rotated in the y-axis by 45°. The α/β TM domains (modeled after the NMR structure of αIIbβ3 TM domains [46]) are displayed for illustrative purposes only, and were not used in the orientation search.

We analyzed our FLIM data by systematically evaluating atomic models to determine the relative orientation of the integrin ectodomain to the membrane plane. The lipid bilayer is modeled as a plane (see methods), with the FLIM distance taken as the shortest distance from the centroid to any point on the plane. Orientation of the integrin on the lipid bilayer requires specifying two angles, the angle between the long axis of Calf-1/Calf-2 domains and the membrane plane (the tilt angle, Θ, being 0° or 90° when the long axis of Calf-2 is either flush with or perpendicular to the membrane, respectively) and the direction of the tilt angle (Φ). With only a single FLIM distance measurement, a large number of combinations of the two angles may be found. Our measured mean FLIM distance of 71Å between Fab107 and the lipid headgroups provided the initial constraint. To determine the angles that satisfy this distance, an atomic model of the integrin/Fab107 complex generated by the fit to the EM data (Figure 3B) is rotated along all available angles and the resulting distance between the Fab107 centroid and the membrane plane measured at each point. There is not as yet an X-ray structure of any intact integrin, and this has compelled our modeling to be performed with ectodomain structures instead, all of which adopt similar bent conformations, where the legs are bent back to position the membrane junctional regions proximal to the head domain. Current structural investigations on full-length integrins lack atomic (or even near-atomic) resolution but do agree that the full-length structure of the inactive conformation is also compact, with its ectodomain likely bent [28], [29]. As a result, our ectodomain model was rotated through a full 360° in Φ and 180° in Θ, which allows the leg domains to vary from completely vertical to lying flat on the surface of the membrane to being buried vertically in the membrane. To check orientations for clashes with the membrane, the membrane was modeled as a simple plane in x-y, and excluding models where any atom dipped below it. A 5° search for both angles generated a total of 114 possible orientations where the CD11b/CD18-Fab107 complex would not clash with the membrane (Figure 5A,B). Applying the further FLIM distance constraint of 71Å between Fab107 and membrane plane further reduced the number of orientations to eight. These solutions possess a range of tilt angles (Θ) of 50° to 60° and an average angle of 52.5°. To check our ectodomain model, we considered the CD11c/CD18 X-ray structure, which does not fit well with our EM ectodomain structure but which might represent the intact full-length structure. This latter model, generated by aligning the αA domains in the CD11c/CD18 and CD11bA/Fab107 X-ray structures, failed to produce any orientation that simultaneously satisfied the membrane clash and FLIM distance criteria.

## Discussion and Conclusions

In this report, we have used electron microscopy and 3D image reconstruction to determine the 3D structure of inactive CD11b/CD18 ectodomain and position its ligand-binding CD11bA domain within the integrin head. We have also utilized FLIM, using the structurally defined reporter of the MIDAS face, Fab107, to precisely define the relation of the CD11bA MIDAS face to the plasma membrane in live cells. The measured distance suggests that the MIDAS face in the cellular integrin is not occluded by the plasma membrane as commonly depicted, but is accessible to soluble macromolecular ligands, while the EM reconstruction indicates that this would be true for this integrin in its bent conformation.

Previous crystal structure work on the related integrin CD11c/CD18 ectodomain indicated that the connection between the ligand-binding αA (CD11cA) domain and the Propeller domains is highly flexible [17]: three of the four molecules within the unit cell in the X-ray structure possessed no density for CD11cA, while the visible CD11cA are stabilized with crystal contacts from symmetry-related molecules (Figure 3E,F). In contrast, our 3D EM structure reveals that CD11bA is fairly well constrained to a small range of conformations in the ectodomain in solution. CD11bA is clearly visible in all of the reference-free class averages (Figure 1A), consistent with a previous 2D EM study in the related CD11c/CD18 ectodomain [30], whereas if the domain had been very flexible, we would expect the density to be averaged out. It is also unlikely that the differences observed between the two β2 integrins relate to the allosteric effect of the introduced disulfide bond that covalently links the α/β heterodimer, as this bond was present at the identical position in both integrin ectodomains.

The EM averages of the CD11b/CD18-Fab107 complex further restricted the range of possible orientations of CD11bA, as evidenced by the clear and unambiguous density for the Fab in the averages (Figure 3). And in contrast with the X-ray structure of CD11c/CD18 ectodomain, CD11bA has been rotated on axis about 35° in our EM structure (Figure 3). One interpretation for these differences is that αA is more flexible in integrin CD11c when compared to that of CD11b. However, the N- and C-terminal aminoacid sequences linking αA to the respective CD11c and CD11b Propeller domains are highly conserved (The N-terminal sequence starting at residue 127 is PRQ in CD11c vs. PQE starting at residue 129 in CD11b while the C-terminal linker is 319IEGTQTGSSSSFEHEMS vs. 317IEGTETTSSSSFELEMA for CD11c and CD11b, respectively). An alternative explanation is that the difference might be due to unfavorable crystal contacts in the CD11c/CD18 ectodomain crystal structure (Figure 3E,F), stabilizing the CD11cA domain in a non-physiologic orientation. The position of CD11bA as defined by the 3D EM structure would allow for an unobstructed movement for the 10Å-displacement of its c-terminal helix 7 associated with ligand-occupancy [31], permitting Glu320 at the end helix 7 to engage the βA domain MIDAS site [15], without additional rotations of the αA domain, thus allowing for the ligand-associated protein movements with only relatively small changes in this domain.

As our acceptor fluorophore is confined to the outer leaflet of the plasma membrane, the FLIM lifetime measurements conducted in the presence of Ca^2+^, provide an accurate distance separating the macromolecular ligand mimetic Fab107 and the membrane. The mean separation between the CD11bA-bound Fab107 and the cell membrane in inactive full-length CD11b/CD18 was approximately 70Å (Figure 5A,B). This is not consistent with an extended model for the integrin [32], where the distance from the membrane to CD11bA would be closer to 160Å, and suggests a much more compact conformation for the inactive state. However, the ∼70Å distance is significantly greater than conventional models of the inactive membrane-bound integrin that rely on depicting the bent configuration of the ectodomain with the long axis of Calf-1/Calf-2 domains aligned perpendicular to the membrane plane [29]. Indeed, the ∼70Å distance places the MIDAS face of CD11bA well clear of the membrane, where it is able to engage macromolecular ligands in solution.

The FLIM distance of the anti-αV mAb Fab17E6 from the plasma membrane for the αA-lacking integrin αVβ3 was previously determined, which yielded a tilt angle of ∼30° [4]. Applying the same nonbiased approach reported here through exploration of the full Euler angle space possible between the X-ray structures and a plane representing the membrane (Figure S2), yielded a range of tilt angles between 30° to 45°. Of interest is that the direction of the tilt (Φ) was remarkably homogenous for both the αA-containing CD11b/CD18 and lacking (αVβ3) integrins, with angles (based on our initial orientation) of 323.6° (S.D. 14.6°) for CD11b/CD18 and 322.2° (S.D. 17.1°) for αVβ3 (this angle determines which face of the integrin is tilting towards the membrane). The virtual identity for the determined tilt ranges indicates continuity in tilting of integrins, suggesting that there is likely to be some structural conservation in the short α/β linker regions with the transmembrane domains such that the link will always ‘hinge’ in the same direction. The two integrins do, however, have different tilt angles (Θ): a mean 34.4° (S.D. 4.2°) tilt for αVβ3 but a 52.5° (S.D. 3.8°) tilt for the αA-containing CD11b/CD18 (Figure S3). Despite the rigorous exploration of the full Euler angle space, none of the allowed orientations will simultaneously satisfy both FLIM distance constraints. This might well represent a real difference between the structures of β2 and β 3 integrins, where the αA-containing β2 integrins would adopt a more obtuse angle relative to the membrane normal. Such an angle would position the αA MIDAS face somewhat more elevated above the surface of the membrane, which might in turn facilitate its binding to macromolecular ligands.

Our current model of an inactive full-length bent integrin embedded a lipid bilayer, if extended to other integrins, would allow unhindered access of Fab fragments of a number of mAbs (with known crystal structures complexed with extracellular integrin domains [3], [33]–[35]) to the cellular integrin (Figure S4). The sole exception is the mAb SG/19, which binds to inactive α5β1 head [36], where the bound Fab would clash with the membrane surface in our full-length integrin model (Figure S4). Binding of this Fab would be allowed if the tilt angle is increased to between 75–80°, although it would no longer be possible to satisfy the FLIM distances for Fab 107 (Figure 5) or for 17E6 Fab (Figure S2). It might also be, as the authors suggest, that the inactive α5β1 ectodomain may not adopt the compact bent conformation found in the other ectodomains with determined crystal or EM structures.

The present data have some bearing on existing models of inside-out integrin activation. In the absence of a crystal structure of a full-length inactive integrin, its Calf-1/Calf-2 domains were empirically positioned orthogonal to the cell membrane in the bent state (see for example [29]), an orientation that sterically blocks the βA MIDAS in the αA-lacking integrin subgroup by the plasma membrane, and places the extra αA domain in the αA-containing subgroup at or within the lipid bilayer. This depiction provided the rationale for the proposed requirement for an inside-out activation-triggered linearity at the genu as a prerequisite for access of extrinsic ligands to the integrin (the switchblade model) [29], [37]. In favor is a negative-stain 2D EM study of αIIbβ3 embedded in small bilayer ‘minidiscs’ [29], which found that in the presence of talin and ligand, 22% of the lipid-reconstituted unliganded αIIbβ3 assumed an extended form [37]. However, the presence of ligand in the sample leaves open the possibility that the extended state is the outcome of conformational memory induced by the prior encounter of the integrin with ligand [38]–[40]. The data presented in this paper using a combination of biophysical methods suggest that the ligand binding site is accessible to extracellular ligands in the bent conformation, providing structure-based support for a number of biochemical studies showing that genu-linearity does not necessarily antecede ligand binding [9], [20], [41]–[44] at least in β2 and β3 integrins as initially proposed by the βTD-centric deadbolt model [45].

## Supporting Information

Figure S1 **Binding of mAb107 to the wild-type and I316G mutant cellular CD11b/CD18, expressed as a percentage of binding of the heterodimer-specific mAb IB4.** Histograms (mean+SD, n = 3) showing binding of mAb 107 to wild-type or I316G CD11b/CD18 stably expressed on K562 in presence of 1 mM Ca^2+^ or 5 mM Mg^2+^/1 mM EGTA. mAb 107 recognizes either integrin in 1 mM Ca^2+^ (low affinity) but not in Mg^2+^/EGTA (high affinity) state.
(TIF)

Figure S2 **A systematic orientation search of αVβ3 ectodomain relative to membrane, employing previously published FLIM data (see text).** A and B, integrin α- (blue), β-(red) subunits and Fab17E6 (yellow) subunits are displayed as a ribbon diagram. A section of a model DMPC membrane is shown as a wire diagram. The ectodomain is sequentially rotated identically to that in Figure 5A,B. The model is again evaluated for clashes with the membrane for each orientation. Shown in cyan are the centroids for Fab17E6 for all of the allowed orientations. Only one of the allowed orientations is displayed. The centroid for the model is indicated by the cyan line, which connects the centroid to the membrane plane. The distance indicated is compatible with the measured FLIM distance. The α/β TM domains (modeled after the NMR structure of αIIbβ3TM domains) are displayed for illustrative purposes only, and were not used in the orientation search. A section of a model DMPC membrane is shown as a wire diagram.
(TIF)

Figure S3 **Plot of the allowed Euler angles for full-length integrin αVβ3 bound to Fab17E6 (open circles) and full length CD11b/CD18 bound to Fab107 (closed circles).** Angles represent the variation at 5 intervals of the ectodomain orientation relative to the transmembrane domains. For each angle pair, the model was checked for steric clashes with the modeled membrane and that the distance of the respective Fab centroid to the membrane corresponded to the value determined by FLIM. Although the orientations do not overlap, they occupy a fairly narrow zone in space.
(TIF)

Figure S4 **Binding of mAb Fab fragments directed against four different αA-lacking integrins as defined by X-Ray crystal structure determination of integrin-Fab complexes (see text).** Ribbon diagrams of αVβ3 ectodomain (α- and β-subunits in blue and red, respectively) complexed to 17E6 Fab (yellow); αIIbβ3 headpiece/10E5 Fab (gray) complex (2vdn.pdb); α4β7 headpiece/ACT-1 Fab (brown) complex (3v4p.pdb), and α5β1 headpiece/SG/19 Fab (green) complex. The ADMIDAS metal ion (cyan sphere), and Propeller and α-genu metal ions (orange spheres) in αVβ3 ectodomain are shown. The diagram was generated by superposing structure of the Propeller domain from the integrin in each complex onto that of the αV structure, using Matchmaker in Chimera. The α/β TM domains (modeled after the NMR structure of αIIbβ3 TM domains) are displayed for illustrative purposes only. A section of a model DMPC membrane is shown as a wire diagram.
(TIF)
