# Supplementary figures and images for: EM Structure of the Ectodomain of Integrin CD11b/CD18 and Localization of Its Ligand-Binding Site Relative to the Plasma Membrane
Source: PLoS One. 2013 Feb 28;8(2):e57951. doi: 10.1371/journal.pone.0057951 (PMC3585415; doi:10.1371/journal.pone.0057951)

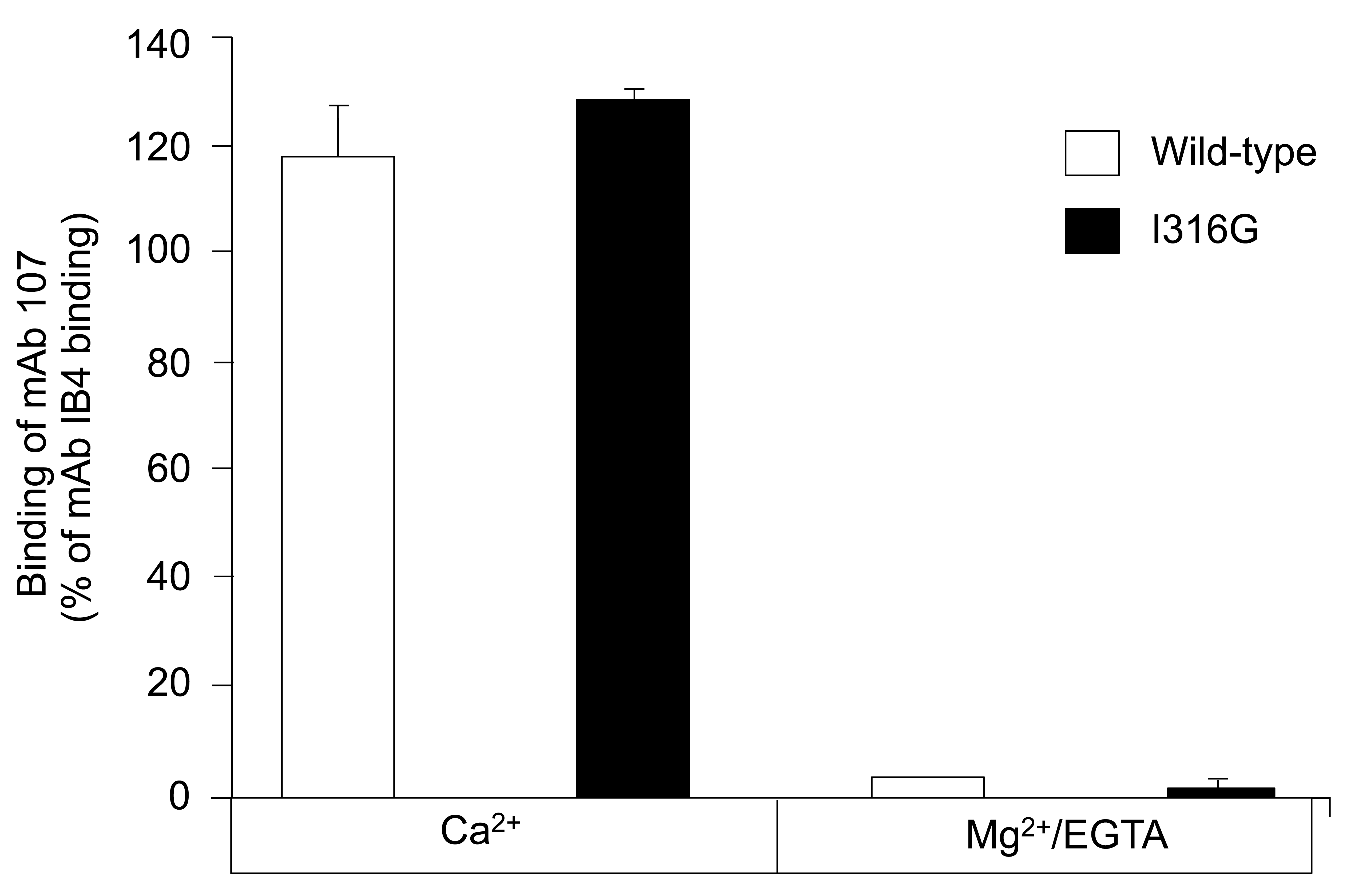

Supplement: Figure S1 — Binding of mAb107 to the wild-type and I316G mutant cellular CD11b/CD18, expressed as a percentage of binding of the heterodimer-specific mAb IB4. Histograms (mean+SD, n = 3) showing binding of mAb 107 to wild-type or I316G CD11b/CD18 stably expressed on K562 in presence of 1 mM Ca2+ or 5 mM Mg2+/1 mM EGTA. mAb 107 recognizes either integrin in 1 mM Ca2+ (low affinity) but not in Mg2+/EGTA (high affinity) state. (TIF) [file pone.0057951.s001.tif]

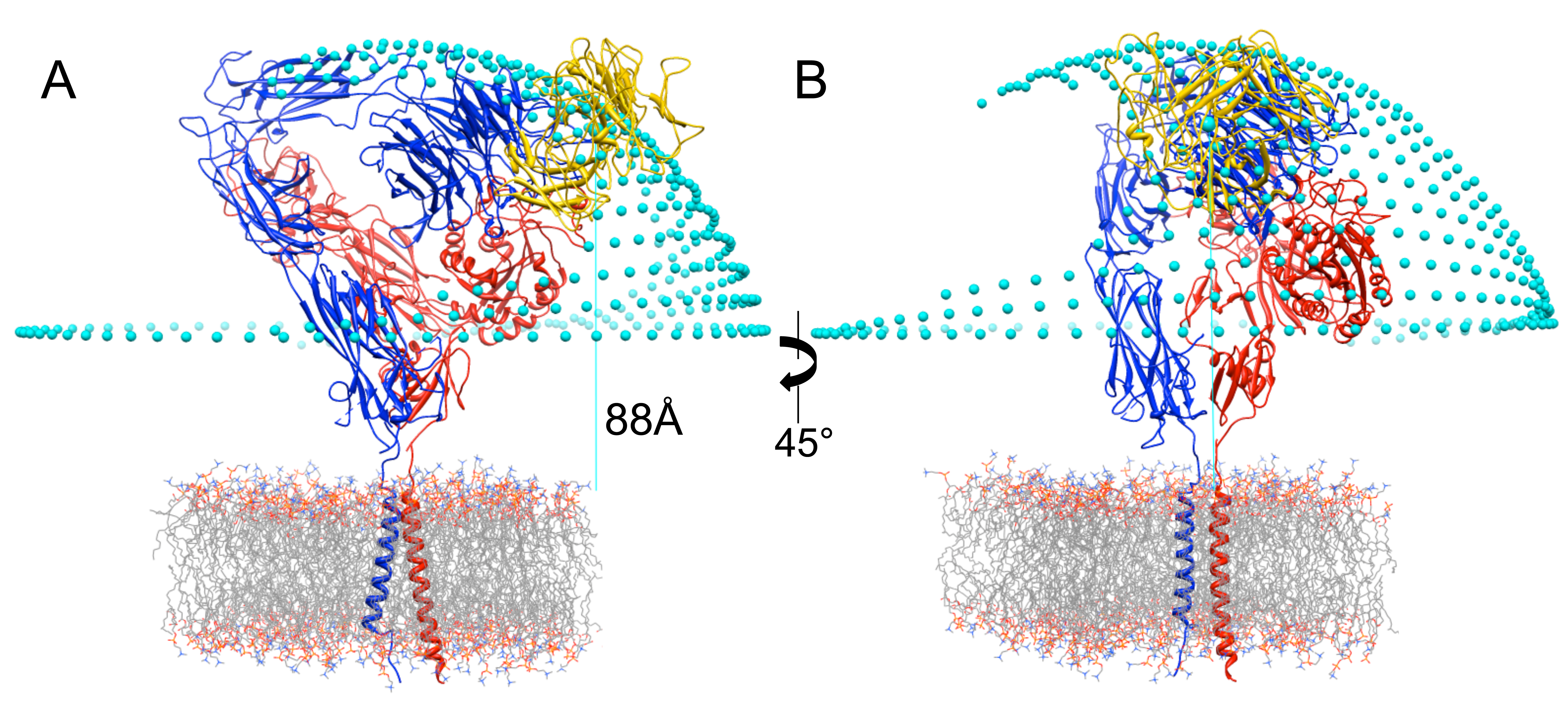

Supplement: Figure S2 — A systematic orientation search of αVβ3 ectodomain relative to membrane, employing previously published FLIM data (see text). A and B, integrin α- (blue), β-(red) subunits and Fab17E6 (yellow) subunits are displayed as a ribbon diagram. A section of a model DMPC membrane is shown as a wire diagram. The ectodomain is sequentially rotated identically to that in Figure 5A,B. The model is again evaluated for clashes with the membrane for each orientation. Shown in cyan are the centroids for Fab17E6 for all of the allowed orientations. Only one of the allowed orientations is displayed. The centroid for the model is indicated by the cyan line, which connects the centroid to the membrane plane. The distance indicated is compatible with the measured FLIM distance. The α/β TM domains (modeled after the NMR structure of αIIbβ3TM domains) are displayed for illustrative purposes only, and were not used in the orientation search. A section of a model DMPC membrane is shown as a wire diagram. (TIF) [file pone.0057951.s002.tif]

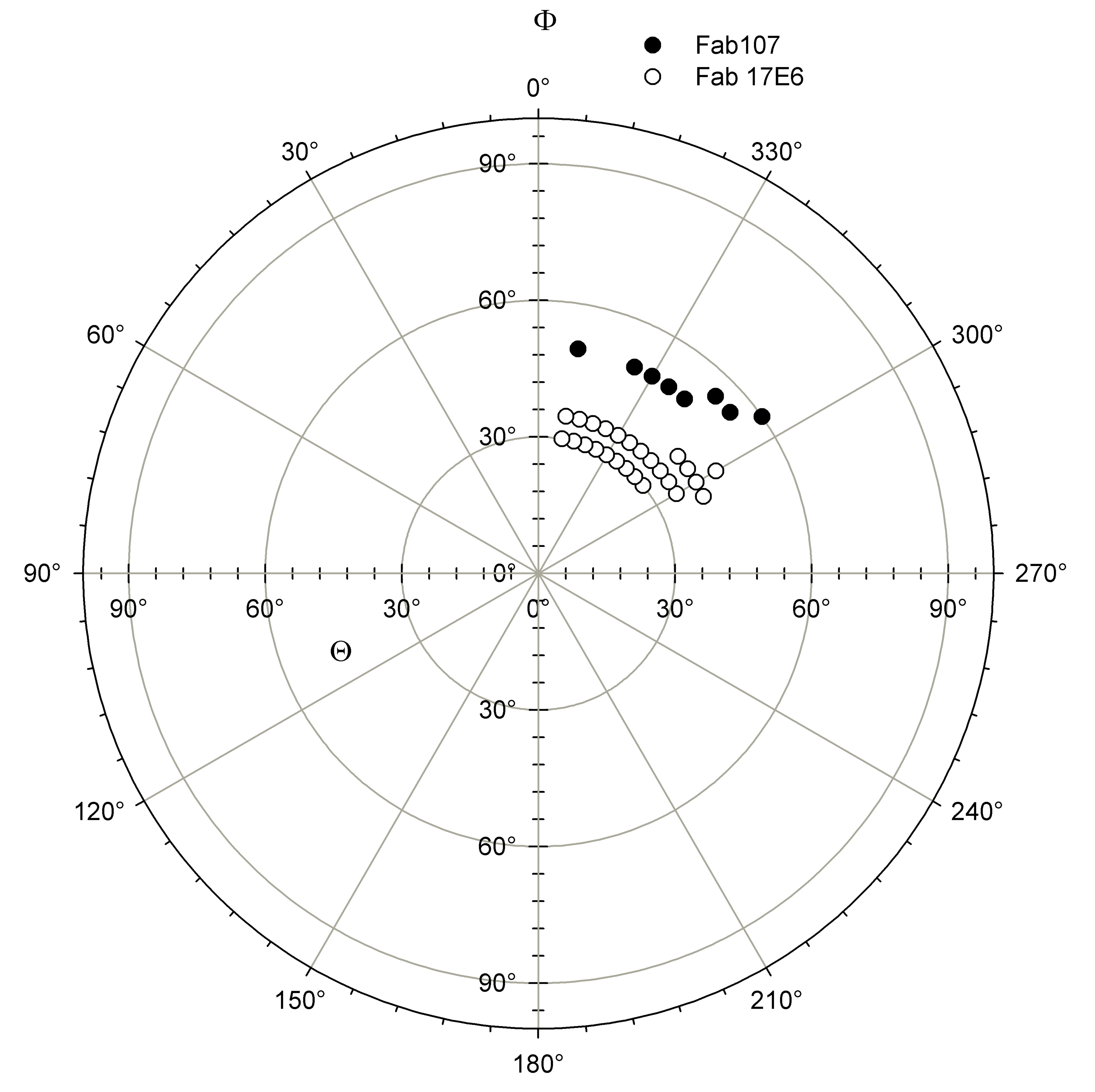

Supplement: Figure S3 — Plot of the allowed Euler angles for full-length integrin αVβ3 bound to Fab17E6 (open circles) and full length CD11b/CD18 bound to Fab107 (closed circles). Angles represent the variation at 5 intervals of the ectodomain orientation relative to the transmembrane domains. For each angle pair, the model was checked for steric clashes with the modeled membrane and that the distance of the respective Fab centroid to the membrane corresponded to the value determined by FLIM. Although the orientations do not overlap, they occupy a fairly narrow zone in space. (TIF) [file pone.0057951.s003.tif]

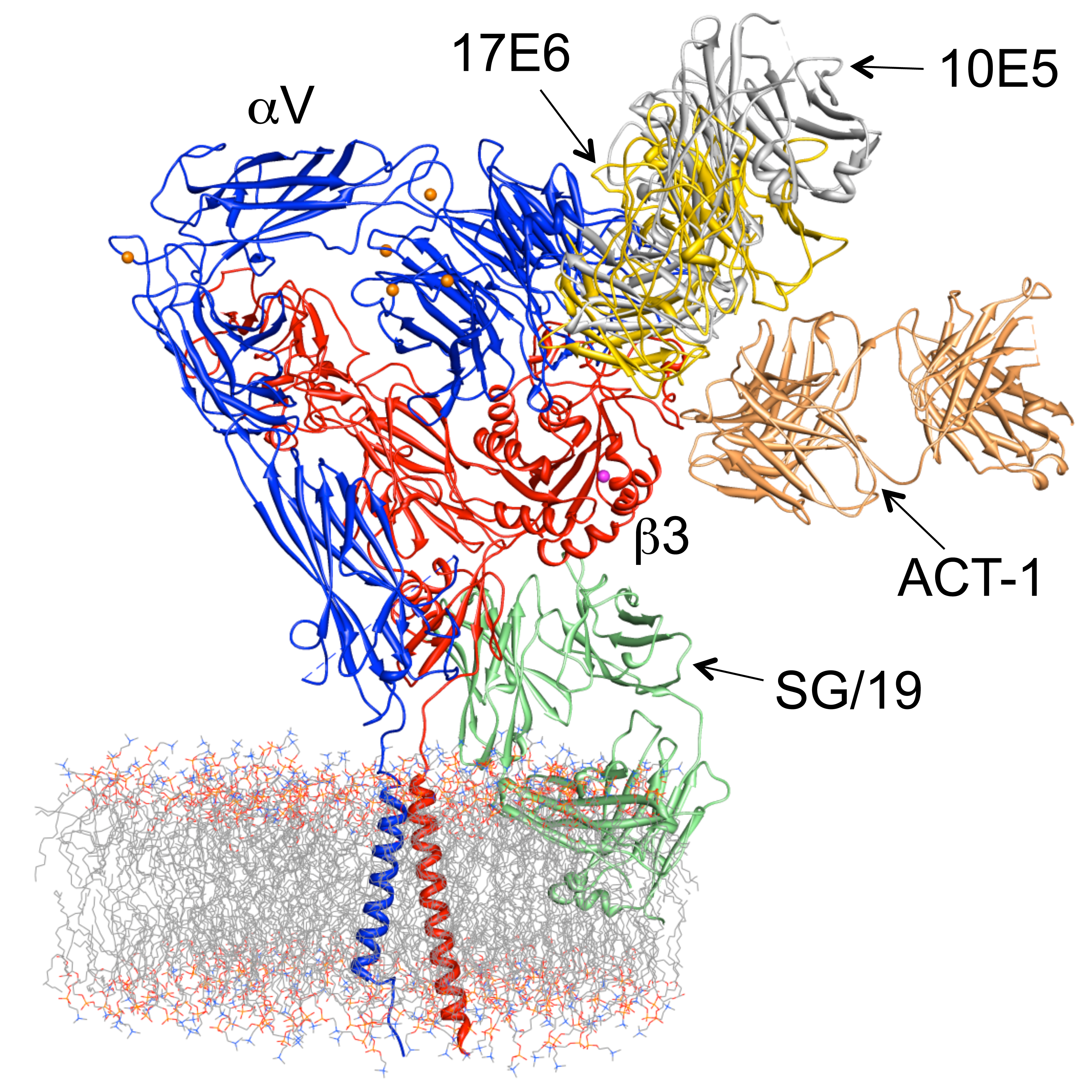

Supplement: Figure S4 — Binding of mAb Fab fragments directed against four different αA-lacking integrins as defined by X-Ray crystal structure determination of integrin-Fab complexes (see text). Ribbon diagrams of αVβ3 ectodomain (α- and β-subunits in blue and red, respectively) complexed to 17E6 Fab (yellow); αIIbβ3 headpiece/10E5 Fab (gray) complex (2vdn.pdb); α4β7 headpiece/ACT-1 Fab (brown) complex (3v4p.pdb), and α5β1 headpiece/SG/19 Fab (green) complex. The ADMIDAS metal ion (cyan sphere), and Propeller and α-genu metal ions (orange spheres) in αVβ3 ectodomain are shown. The diagram was generated by superposing structure of the Propeller domain from the integrin in each complex onto that of the αV structure, using Matchmaker in Chimera. The α/β TM domains (modeled after the NMR structure of αIIbβ3 TM domains) are displayed for illustrative purposes only. A section of a model DMPC membrane is shown as a wire diagram. (TIF) [file pone.0057951.s004.tif]
